# Presentation, management and mortality after a first MI in people with and without asthma: A study using UK MINAP data

**DOI:** 10.1177/1479972317702140

**Published:** 2017-04-10

**Authors:** Paulo Pinto, Kieran J Rothnie, Kelvin Lui, Adam Timmis, Liam Smeeth, Jennifer K Quint

**Affiliations:** 1 Faculty of Epidemiology and Population Health, London School of Hygiene and Tropical Medicine, London, UK; 2 National Heart and Lung Institute, Imperial College London, London, UK; 3 Faculty of Life Sciences, University College London, London, UK; 4 Barts NIHR Biomedical Research Unit, Queen Mary University of London, London, UK

**Keywords:** Asthma, cardiovascular disease, mortality, epidemiology, quality of care, myocardial infarction

## Abstract

Asthma has been associated with a higher incidence of myocardial infarction (MI), higher prevalence of MI risk factors and higher burden of cardiovascular diseases. However, detailed associations between the presentation and initial management at the time of MI and post-MI outcomes in people with asthma compared to the general population have not been studied. A total of 300,161 people were identified with a first MI over the period 2003–2013 in the Myocardial Ischaemia National Audit Project database, of whom 8922 (3%) had asthma. Logistic regression was used to compare presentation, in-hospital care, in-hospital and 180-day post-discharge all-cause mortality in people with and without asthma adjusting for demographics and comorbidities, diagnosis on arrival and secondary prevention. People with asthma were more likely to have a delay in their MI diagnosis following an STEMI (ST-elevation myocardial infarction; odds ratio (OR) 1.38, confidence interval CI 1.06–1.79) but not an nSTEMI (non-ST-elevation myocardial infarction; OR 1.04, CI 0.92–1.17) compared to people without asthma and a delay in reperfusion (OR 1.19, CI 1.09–1.30) following an STEMI. They were much less likely to be discharged on a beta blocker following an STEMI or nSTEMI (OR 0.24, CI 0.21–0.28 and OR 0.27, CI 0.24–0.30, respectively). There was no difference in in-hospital or 180-day mortality (OR 0.98, CI 0.59–1.62 and OR 0.99, CI 0.72–1.36) following an STEMI or nSTEMI (OR 0.89, CI 0.47–1.68 and OR 1.05, CI 0.85–1.28). Although people with asthma were more likely to have a delay in diagnosis following an STEMI but not an nSTEMI compared to the general population, were more likely to have a delay in reperfusion therapy and were much less likely to receive beta blockers following an STEMI or nSTEMI, there was no difference in the prescriptions of other secondary prevention medications. None of the differences in presentation or management were associated with an increase in all-cause in-hospital or 180-day mortality in people with asthma compared to the general population.

## Introduction

Asthma is a chronic inflammatory disease of the airways associated with local hyper-responsiveness and infiltration of immune system cells.^1,2^ Worldwide, asthma is estimated to affect between 240 million and 315 million people, affecting adult women more than men.^3,4^ In England alone, asthma affects over 5.6 million people, representing a challenge to both quality of life and health services costs.^5^

Coronary artery disease is a major cause of death worldwide, and despite declining age-specific mortality rates in developed countries, a shift towards ageing populations maintains growing absolute numbers.^6^ In the United Kingdom, the incidence of myocardial infarction (MI) in 2009 was estimated as 255 events/100,000 person-years for men and 113 events/100,000 person-years for women, being responsible for over 18% of male and 13% of female deaths.^7^

Previous studies have shown that the burden of ischemic heart disease is higher in people with asthma compared to the general population with increased risk varying from 40% to 90%.^8–11^ This increased risk is thought to be based on common pathological characteristics including shared inflammatory pathways between asthma and cardiovascular disease, susceptibility to atherosclerosis and cytokine upregulation.^12–14^ People with asthma also commonly have comorbidities considered risk factors for MI, such as obesity, depression, anxiety and chronic obstrcutive pulmonary disease COPD.^15–17^ A cohort study investigating comorbidities in people with asthma in the UK primary care estimated that they have 3.5 times the risk of having any cardiac comorbidity diagnosed compared to the general population.^18^ More recently, two cross-sectional studies found a higher prevalence of angina, hypertension, stroke and heart failure among people with asthma, with associations overall stronger for the elderly and for women.^19,20^ Although less evidence exists around mortality, previous studies have shown a higher proportion of ischaemic and non-ischaemic deaths among asthmatics compared to the general population, with cardiovascular disease estimated as a major cause of mortality among the elderly.^21^

Despite the increased risk of cardiovascular disease, no published study to date has investigated diagnosis and management of post-MI comparing people with and without asthma. We have recently investigated differences in presentation, diagnosis and management of MI in people with and without COPD.^22^ We found that people with COPD had higher mortality post-MI compared to the general population and that this might be explained in part by differences in recognition and management of MI. Therefore, we hypothesised that the same may be true for people with asthma. Using the Myocardial Ischaemia National Audit Project database (MINAP),^23^ we investigated the differences in presentation, management and prognosis following a first MI in people with and without asthma.

## Methods

### Data source

MINAP is a registry of all admissions for MI and other acute coronary syndromes (ACSs) to hospitals in the United Kingdom from 2003. The data set includes information on patient demographics, comorbidities, drugs on admission, initial diagnosis, final diagnosis, in-hospital drug treatment, timing of reperfusion therapies, in-hospital outcome and drugs given on discharge.^9^

### Study population

**Figure 1.**
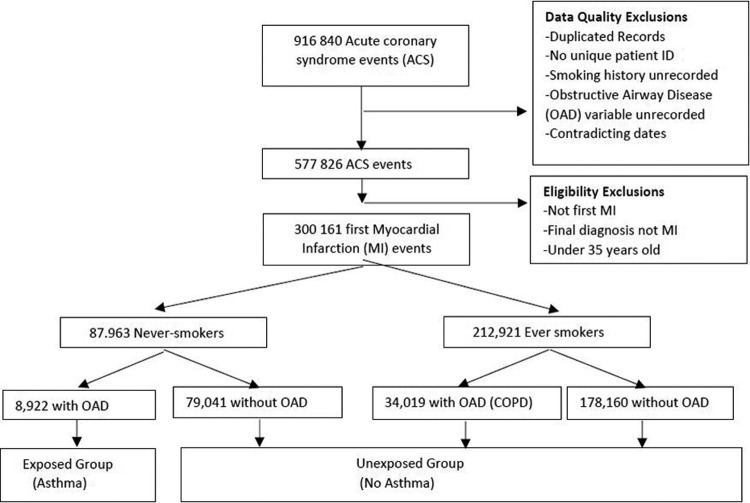
Patient selection flowchart.

All patients with a first diagnosis of ST-elevation myocardial infarction (STEMI) from January 2003 to June 2013 or non-ST-elevation myocardial infarction (nSTEMI) from January 2004 to December 2012 were included. Records were excluded where there was not a unique patient identifier; if there were missing values for presence of obstructive airway disease (OAD) or smoking history; or if Office of National Statistics (ONS) mortality data were missing (Figure 1).

### Exposure definition

The exposure of interest was asthma. The MINAP database does not collect asthma status directly but the presence of self-reported OADs, which may be either asthma or COPD, is recorded. To differentiate patients with asthma and COPD, those with asthma were defined as those with OAD who never smoked, whereas COPD patients were considered ex-smokers or current smokers. This approach was recently used in a similar study investigating COPD patients in the same data set and has been used in other studies.^22,23^ The validity of this method in MINAP was ascertained through a linkage of a subset of data between MINAP and Clinical Practice Research Datalink CPRD, a large UK clinical database of primary medical care involving more than 5 million active patients.^24,25^ In that strategy, COPD patients were identified in MINAP with agreement of 90.9%.

### Presentation

Features including age, sex, ethnicity, type of MI, final diagnosis as STEMI or nSTEMI and comorbidities associated with presentation at the time of admission to hospital were compared between people with and without asthma.

### Recognition and management

Delay in diagnosis of MI, use and time to reperfusion after an STEMI, admission to a coronary care unit (CCU) use of angiography or percutaneous coronary intervention PCI/coronary artery bypass graft CABG in hospital after an nSTEMI and discharge on secondary prevention drugs were investigated in both groups. Two definitions of delay in diagnosis were investigated. For patients with a final diagnosis of STEMI, this was delay in diagnosis of definite STEMI (defined as those who did not have an initial diagnosis of definite STEMI). For those patients with a final diagnosis of nSTEMI, this was delay in diagnosis of ACS (defined as those whose initial diagnosis was not STEMI, probable MI or ACS). Delay in reperfusion was defined as any fibrinolysis done 30 minutes after diagnosis or any primary percutaneous coronary intervention pPCI done 90 minutes after diagnosis.^26^

### Mortality outcomes

We investigated in-hospital and 180-day post-discharge all-cause mortality. In-hospital mortality is compulsorily recorded in MINAP. The UK ONS collects data on all recorded deaths in England and Wales.^27^ MINAP is linked with ONS mortality data, which provides data on vital status at 180 days post-discharge. Mortality at 180 days post-discharge was assessed for those who survived until discharge.

### Statistical analyses

The baseline characteristics (demographics and comorbidities) were tabulated for people with and without asthma. Logistic regression was used to control for age and gender differences. The main analysis was conducted separately for STEMIs and nSTEMIs. The same approach was used for management and mortality outcomes. First, people with and without asthma were compared in each outcome using percentages and percentage differences. Then, logistic regression was used to calculate crude odds ratios (ORs). Sequentially, we added age, sex, smoking status and comorbidities to the models.

To decide which comorbidities would be included in the full model, conceptual frameworks were designed and variables determined a priori as possible confounders were preselected, independently of a statistical significant association with exposure or outcome. The comorbidities: diabetes, cerebrovascular disease, chronic kidney disease, congestive cardiac failure, previous angina, hypertension, treatment of hypercholesterolemia, treatment with antiplatelet therapy, previous PCI or previous CABG, tachycardia at admission and hypotension at admission were selected based on prediction scores for MI management and mortality.^28–30^ Obesity was not included as this variable was poorly recorded.

### Missing values

Missing values were not imputed for demographic characteristics. Missing values for comorbidities collected as categorical variables were imputed following the suggested approach for MINAP, imputing unrecorded as ‘no’ or ‘absent’. Comorbidities collected as quantitative and analysed as categorical variables using a threshold (e.g. obesity) were not imputed, and only complete case records were analysed. Discharge medications were imputed as per the comorbidities. For the other outcomes, only complete case records were analysed.

### Sensitivity analysis

A sensitivity analysis was conducted restricting the analysis to never smokers only to account for eventual unaccounted confounding by smoking status.

### Ethics

This study was approved by LSHTM MSc Research Ethics Committee (9719) and the MINAP academic group (13-MNP-07).

## Results

### Presentation

A total of 300,161 patients were identified with a first MI over the study period (2003–2013), of whom 8922 (3%) had asthma. On presentation with an MI, people with asthma were older than those without (mean age 72.8 vs. 67.3), were more likely to be female (54.4% vs. 32.5%) and had a higher proportion of nSTEMIs than STEMIs (65.5% vs. 53.9%, OR 1.29, 95%CI 1.23–1.35). By definition, all people with asthma were never smokers compared to 27.1% of non-asthmatics.

After controlling for age, sex and smoking status, people with asthma were more likely to have had previous angina (OR 1.22, CI 1.16–1.29), tachycardia at admission (OR 1.41, CI 1.33–1.49), chronic kidney disease (OR 1.34, CI 1.22–1.49), congestive cardiac failure (OR 1.81, CI 1.65–1.99), diabetes (OR 1.12, CI 1.06–1.18) and peripheral vascular disease (OR 1.18, CI 1.03–1.35). They were less likely to have a family history of MI (OR 0.93, CI 0.88–0.99) or hyperlipidaemia (OR 0.92, CI 0.87–0.97; Table 1).

**Table 1.** Comparison on demographics and comorbidities between people with and without asthma.

Total patients 300,161 (100%)	No asthma 291,239 (97%)	Asthma 8922 (3%)	
Baseline characteristics			OR adjusted for age, sex and smoking (no asthma as baseline)
Age			
35–50	38,273 (13.1%)	579 (6.5%)	
51–60	58,603 (20.1%)	1018 (11.4%)	
61–70	70,135 (24.1%)	1831 (20.5%)	
71–80	69,644 (23.9%)	2826 (31.7%)	
81 and older	54,421 (18.7%)	2261 (29.8%)	
Mean age	67.3	72.8	
Gender (*n* = 299,125)			
Male	195,648 (67.4%)	4053 (45.5%)	
Female	94,579 (32.5%)	4845 (54.4%)	
Ethnicity (*n* = 167,261)			
White	153,093 (94.3%)	4508 (89.2%)	
Non-White	9114 (5.6%)	546 (10.8%)	
Smoking status			
Never	79,041 (27.1%)	8922 (100.0%)	
Ex-smoker	107,277 (36.8%)	0	
Current	104,291 (36.0%)	0	
Type of MI			
STEMI	133,990 (46.0%)	3076 (34.4%)	1 (reference)
nSTEMI	157,249 (53.9%)	5846 (65.5%)	1.29 (1.23–1.35)
Previous angina	46,975 (16.1%)	1886 (21.1%)	1.22 (1.16–1.29)
Previous PCI	7371 (2.5%)	236 (2.6%)	1.03 (0.90–1.18)
Previous CABG	6309 (2.1%)	187 (2.1%)	0.92 (0.79–1.07)
Diabetes (*n* = 296,535)	43,737 (15.2%)	1658 (18.8%)	1.12 (1.06–1.18)
Familial history of MI (*n* = 218,072)	73,986 (34.9%)	1735 (27.5%)	0.93 (0.88–0.99)
Treated for Hypertension	128,756 (44.2%)	4,432 (39.6%)	0.96 (0.92–1.00)
Treated for hyperlipidaemia	80,479 (27.63%)	2262 (25.3%)	0.92 (0.87–0.97)
Treated with antiplatelets	65,116 (22.2%)	2195 (24.6%)	1.01 (0.96–1.07)
Peripheral vascular disease	10,776 (3.7%)	248 (2.8%)	1.18 (1.03–1.35)
Cerebrovascular disease	18,959 (6.5%)	688 (7.7%)	1.01 (0.93–1.10)
LVEF left ventricular ejection fraction<30% (*n* = 99,198)	9499 (9.8%)	281 (10.2%)	0.98 (0.86–1.12)
Chronic kidney disease	9648 (3.3%)	464 (5.2%)	1.34 (1.22–1.49)
Congestive cardiac failure	8894 (3.0%)	573 (6.4%)	1.81 (1.65–1.99)
Hypotension at admission (*n* = 271,974) systolic blood pressure SBP <90 mmHg	9161 (3.5%)	247 (3.0%)	0.85 (0.74–0.98)
Tachycardia at admission (*n* = 272,761) heart rate HR >100 BPM	77,522 (29.3%)	3140 (38.6%)	1.41 (1.33–1.49)

OR: odds ratio; STEMI: ST-elevation myocardial infarction; nSTEMI: non-ST-elevation myocardial infarction; MI: myocardial infarction.

### In-hospital management

A total of 137,066 patients were identified with an STEMI, of whom 3076 (2.2%) were asthmatics. After adjustment for age, sex, smoking status, calendar year and comorbidities, people with asthma were more likely to have a delay in their diagnosis (OR 1.38, CI 1.06–1.79), a delay in reperfusion (OR 1.19, CI 1.09–1.30), however were not less likely to have reperfusion therapy at all compared to those without asthma (OR 0.93, CI 0.79–1.06). After adjusting for potential confounders, those with asthma were less likely to be admitted to a CCU following an nSTEMI (OR 0.90, CI 0.85–0.96) but had the same odds of admission to a CCU following an STEMI (OR 0.98, CI 0.89–1.08). Median time to reperfusion following admission for STEMI was 45.9 minutes interquartile range (IQR, 30.6–74.3) for asthmatics and 43.7 minutes (IQR, 28.4–69.9) for non-asthmatics for those who had pPCI; and 32.8 minutes (IQR, 19.7–74.3) for asthmatics and 28.4 minutes (IQR, 17.5–56.8) for non-asthmatics for those who had thrombolysis. With respect to prescription of secondary preventative medications, people with asthma had 76% lower odds of being discharged with beta blockers in the fully adjusted model (OR 0.24, CI 0.21–0.28; Table 2).

**Table 2.** In hospital management for STEMIs.

	No asthma (*n*,%) 133,990	Asthma (*n*,%) 3076	Total (*n*,%) 137,066	
			Crude OR (95% CI)	Fully^a^ adjusted OR (95% CI)
In-hospital management for STEMIs				
Diagnosis and admission				
Diagnosis delay	*n* = 8261 (6.2%)	*n* = 323 (10.5%)	1.45 (1.34–1.58)	1.38 (1.06–1.79)
Admission to CCU (*n* = 135,927)	*n* = 112,594 (84.7%)	*n* = 2480 (81.3%)	0.79 (0.72–0.86)	0.98 (0.89–1.08)
Invasive procedures				
Delayed reperfusion *n* = 111,470	*n* = 42,425 (38.9%)	*n* = 1047 (45.2%)	1.30 (1.19–1.41)	1.19 (1.09–1.30)
Use of reperfusion	*n* = 106,207 (79.3%)	*n* = 2231 (72.5%)	0.69 (0.64–0.74)	0.93 (0.79–1.10)
Use of pPCI	*n* = 47,382 (35.4%)	*n* = 931 (30.27%)	0.79 (0.73–0.86)	0.92 (0.8–1.06)
Secondary prevention				
Discharge on beta blocker BB *n* = 132,588	*n* = 99,175 (74.0%)	*n* = 1399 (45.5%)	0.27 (0.25–0.30)	0.24 (0.21–0.28)
Discharge on aspirin *n* = 132,588	*n* = 110,429 (85.2%)	*n* = 2418 (82.6%)	0.84 (0.80–0.88)	0.97 (0.78–1.21)
Discharge on clopidogrel *n* = 132,588	*n* = 83,102 (64.1%)	*n* = 1785 (61.0%)	0.87 (0.83–0.90)	1.05 (0.87–1.27)
Discharge on statin *n* = 132,588	*n* = 110,382 (85.1%)	*n* = 2431 (83.1%)	0.93 (0.84–1.04)	1.17 (0.92–1.48)
Discharge on angiotensin converting enzyme inhibitor ACEi *n* = 132,588	*n* = 103,602 (79.9%)	*n* = 2253 (77.0%)	0.89 (0.81–0.97)	1.10 (0.91–1.35)

OR: odds ratio; STEMI: ST-elevation myocardial infarction; CCU:coronary care unit; MI: myocardial infarction.

^a^Adjusted for age, sex, smoking, year of admission, diabetes, Cerebrovascular disease CVSD, chronic renal failure CRF, congestive cardiac failure CCF, peripheral vascular disease PVD, low LVEF, previous angina, previous PCI, previous CABG, family history of MI, treatment for hypertension, treatment for hyperlipidaemia, treatment with antiplatelets, tachycardia and hypotension at admission.

A total of 165,095 patients were identified with an nSTEMI, of whom 5846 (3.6%) had a diagnosis of asthma. After adjustment for age, gender, calendar year and comorbidities, there was no evidence of a delay in diagnosis (OR 1.04, CI 0.92–1.17) between people with and without asthma but those with asthma were less likely to have elective PCI/CABG (OR 0.88, CI 0.82–0.95). With respect to secondary prevention, people with asthma had 73% lower odds of being discharged with beta blockers in the fully adjusted model (OR 0.27, CI 0.24–0.30; Table 3). There was no difference in the prescription of other secondary prevention medications.

**Table 3.** In-hospital management for nSTEMIs.

	No asthma (*n*, %) 157,249	Asthma (*n*, %) 5846	Total (*n*, %) 163,095	
			Crude OR (95% CI)	Fully^a^ adjusted OR (95% CI)
In-hospital management for nSTEMIs				
Diagnosis and admission				
Diagnosis delay	*n* = 57,321 (36.5%)	*n* = 2595 (44.4%)	1.39 (1.32–1.46)	1.04 (0.92–1.17)
Admission to CCU *n* = 162,343	*n* = 59,506 (38.0%)	*n* = 1921 (33.0%)	0.80 (0.76–0.84)	0.90 (0.85–0.96)
Invasive procedures				
Elective angiography	*n* = 80,193 (51.0%)	*n* = 2470 (42.3%)	0.70 (0.66–0.74)	0.83 (0.72–0.95)
Elective PCI/CABG	*n* = 37,729 (24.5%)	*n* = 1053 (18.0%)	0.69 (0.65–0.74)	0.88 (0.82–0.95)
Secondary prevention				
Discharge on BB *n* = 159,345	*n* = 96,744 (61.5%)	*n* = 1968 (33.7%)	0.30 (0.28–0.32)	0.27 (0.24–0.30)
Discharge on antiplatelet	81.3%	82.4%	1.07 (1.00–1.15)	1.03 (0.96–1.11)
Discharge on aspirin *n* = 159,345	*n* = 117,257 (76.3%)	*n* = 4262 (75.0%)	0.93 (0.87–0.98)	0.94 (0.82–1.08)
Discharge on clopidogrel *n* = 159,345	*n* = 86,326 (56.2%)	*n* = 3084 (54.2%)	0.92 (0.87–0.97)	1.02 (0.9–1.16)
Discharge on statin *n* = 159,345	*n* = 115,549 (75.2%)	*n* = 4140 (72.7%)	0.91 (0.85–0.97)	0.96 (0.84–1.10)
Discharge on ACEi *n* = 159,345	*n* = 98,429 (64.1%)	*n* = 3521 (61.9%)	0.94 (0.88–0.99)	0.97 (0.85–1.09)

OR: odds ratio; nSTEMI: non-ST-elevation myocardial infarction; CCU:coronary care unit; MI: myocardial infarction.

^a^Adjusted for age, sex, smoking, year of admission, diabetes, CVSD, CRF, CCF, PVD, low LVEF, previous angina, previous PCI, previous CABG, family history of MI, treatment for hypertension, treatment for hyperlipidaemia, treatment with antiplatelets, tachycardia and hypotension at admission.

### Mortality

There were 7165 in-hospital deaths following an STEMI. After controlling for age, sex, smoking status, calendar year and comorbidities, there was no evidence of a difference in in-hospital mortality between people with and without asthma (OR 0.98, CI 0.59–1.62). This was also seen for 180-day post-discharge all-cause mortality (OR 0.99, CI 0.72–1.36; Table 4).

**Table 4.** Mortality following STEMIs.

Asthma status for STEMIs	No asthma (*n*) 133,990	Asthma (*n*) 3076	Total (*n*) 137,066		
Mortality^a^ for STEMIs			Crude OR (95% CI)	OR adjusted for age, sex, smoking and comorbidities^a^ (95% CI)	Fully adjusted OR (95%CI)
In-hospital mortality	*n* = 4329 (3.2%)	*n* = 149 (4.8%)	1.64 (1.43–1.87)	0.98 (0.59–1.63)	0.98 (0.59–1.62)^b^
180-day post-discharge mortality *n* = 132,583	*n* = 7609 (5.9%)	*n* = 282 (9.6%)	1.62 (1.39–1.89)	0.96 (0.71–1.31)	0.99 (0.72–1.36)^c^

STEMI: ST-elevation myocardial infarction; OR: odds ratio; MI: myocardial infarction.

^a^Adjusted for age, sex, smoking, year of admission, diabetes, CVSD, CRF, CCF, PVD, low LVEF, previous angina, previous PCI, previous CABG, family history of MI, treatment for hypertension, treatment for hyperlipidaemia, treatment with antiplatelets, tachycardia and hypotension at admission

^b^Adjusted for ^a^, diagnosis delay and use of reperfusion.

^c^Adjusted for ^b^, beta blocker, aspirin, clopidogrel, statin and ACEi at discharge.

There were 9775 in-hospital deaths following an nSTEMI. There was no difference in in-hospital mortality in people with and without asthma after adjusting for confounders (OR 0.89, CI 0.47–1.68) or in 180-day post-discharge all-cause mortality (OR 1.05, CI 0.85–1.28; Table 5).

**Table 5.** Mortality following nSTEMIs.

Asthma status for nSTEMIs	No asthma (*n*) 157,249	Asthma (*n*) 5846	Total (*n*) 163,095		
Mortality for nSTEMIs			CRUDE OR (95% CI)	OR adjusted for age, sex, smoking and comorbidities^a^ (95%CI)	Fully adjusted OR (95%CI)
In-hospital mortality	*n* = 3597 (2.3%)	*n* = 153 (2.6%)	1.32 (1.20–1.46)	0.89 (0.48–1.68)	0.89 (0.47–1.68)^b^
180-day post-discharge mortality *n* = 159,343	*n* = 17,003 (11.1%)	*n* = 872 (15.3%)	1.44 (1.31–1.57)	1.10 (0.90–1.33)	1.05 (0.85–1.28)^c^

nSTEMI: non-ST-elevation myocardial infarction; OR: odds ratio; MI: myocardial infarction.

^a^Adjusted for age, sex, year of admission, aiabetes, CVSD, CRF, CCF, previous angina, treatment for hypertension, treatment for hypercholesterolemia, treatment with antiplatelets, previous PCI, previous CABG, tachycardia and hypotension at admission.

### Sensitivity analysis

The analysis was repeated restricting the population to non-smokers only. Minimal differences were seen compared to the main analysis (see Tables S1 and S2 for full results of all analyses). In all, 35,775 patients admitted with an STEMI were non-smokers, of whom 3076 (8.6%) had asthma; 52,188 patients admitted with an nSTEMI were non-smokers, of whom 5846 (10.5%) had asthma. There was no difference in in-hospital or 180-day mortality following an STEMI or nSTEMI in this analysis (Tables S1 and S2).

## Discussion

This study investigated differences in presentation, management and mortality following a first MI in people with and without asthma. We found that on admission to hospital following an MI, people with asthma were more likely to be older, female, and have a number of pre-existing cardiovascular comorbidities. Following an STEMI, they were more likely to have a delay in their diagnosis and a delay in reperfusion; however, differences in the median time to reperfusion were very small. Those with asthma were not less likely to receive reperfusion therapy or have PCI. They were less likely to receive a beta blocker on discharge but there was no difference in the prescription of other standard secondary prevention medications on discharge. Following an nSTEMI, people with asthma did not have a delay in diagnosis compared to people without asthma, nor was there any difference in acute in-hospital management. Again they were less likely to receive a beta blocker on discharge but there was no difference in the prescription of other standard secondary prevention medications on discharge. Unlike following an MI in people with COPD,^22^ these differences were not associated with an increase in mortality among people with asthma. Perhaps this is unsurprising as the asthma group was at substantially higher risk in terms of age, congestive cardiac failure, chronic kidney disease and diabetes. These competing risks may be more important in terms of prognosis than prescription of beta blockers and delays in treatment.

The higher prevalence of females and comorbid conditions such as chronic kidney disease, diabetes and congestive cardiac failure among people with asthma was expected, and these associations have been described in other populations.^31–36^ This adds validity to our asthma definition. The lower prevalence of a family history of MI and hyperlipidaemia, both important risk factors for MI, supports the concept that asthma itself may be an independent risk factor for MI.^8–10,20^

We also noted a higher prevalence of tachycardia, at the time of MI in people with asthma compared to those without. It is possible that the higher heart rate among people with asthma may be a consequence of the use of beta-2 agonists used in the treatment of asthma, suggesting that patients might mistake the episode for an asthma acute attack. Equally, the delay in diagnosis of STEMI may arise as healthcare professionals mistake breathlessness for an acute exacerbation of asthma, rather than considering a cardiovascular event. The higher prevalence of nSTEMIs compared to STEMI among people with asthma suggests an impact of respiratory diseases in the pathophysiology of ischemic events, and similar results have been demonstrated for COPD.^22^

Another new finding refers to the influence a delayed diagnosis of an STEMI had on delayed time to reperfusion. Interestingly, though this delay in perfusion was not associated with increased mortality, something which was seen in a COPD population,^22^ and this likely represents the smaller median delay in reperfusion for asthmatics compared to a larger delay for those with COPD. Our study also highlights the potential for patients with an established diagnosis to be admitted to that speciality even when they present with a different problem. This was evident for nSTEMIs but not STEMIs from our results. Following an nSTEMI, those with asthma were less likely to be admitted to CCU. This may be a reason why asthma patients were less likely to receive investigations and treatment such as angiography or PCI following nSTEMI.

Our findings are in keeping with the previous literature; however, many previous studies have focused collectively on OADs (asthma or COPD combined as an exposure),^37–39^ with four having studied COPD patients with a clinical or pharmacological exposure definition, without spirometry or imaging to differentiate between asthma and COPD.^39–42^ In six of seven of these studies, patients with OADs were older and more likely to be female, except Chen et al.^42^ Four studies split the analysis into STEMIs and nSTEMIs; and in all studies, nSTEMIs were more common among people with OADs.^37,38,41,43^ Comorbidities varied more among the studies. Overall, OAD patients were more likely to be hypertensive,^37,39,40,43^ diabetic,^37,38,41,43^ have cardiac failure,^39–41,43^ previous angina^38,39^ and chronic kidney disease.^39–41,43^ They were also admitted with higher mean heart rate^38,41,43^ and one reported lower mean systolic blood pressure.^43^

In-hospital procedures have also been shown to be different among people with and without OADs. In six studies, patients with COPD or asthma were less likely to undergo invasive procedures (pPCI, PCI, CABG and angiography). Two studies investigated delayed reperfusion, but this was not seen to differ between those with and without OADs.^38,43^ All studies reported lower prescription of in-hospital or beta blockers at discharge. In-hospital mortality was investigated in four studies.^38,39,41,43^ In three studies, mortality was higher in those with OAD but only in nSTEMIs.^39,40,43^ Long-term mortality (1 year) was investigated in three studies, and OADs were an independent predictor of higher mortality in all.^37,40,42^

A strength of our study is that the analysis was restricted to the patients without prior infarctions, a common approach to avoid confounding effects of factors related to previous events (e.g. secondary prevention medications, severity and type of infarction, ascertainment of diagnosis, date of the event, etc.). Additionally, having validated a diagnosis of COPD using linked MINAP and primary care data previously,^44^ we were able to identify a population of people with asthma unlike in other studies, allowing investigation in this group alone. However, our study has a number of limitations. Firstly, we were limited in our ability to make a diagnosis of asthma in the MINAP database, and differential misclassification is expected in variables associated with smoking status; particularly demographics and comorbidities (e.g. over-representation of older people among asthmatics). However, sensitivity analyses restricted to non-smokers only were used to assess validity of the results. Secondly, OAD is a self-reported parameter in the MINAP database. It is known that self-reporting of OAD is less sensitive and less specific than spirometry tests, and it is possible this led to some non-differential misclassification, but this would simply lead to a dilution of the results to the null. Finally, 180-day post-discharge mortality is ascertained by linkage of MINAP with ONS which does not include cause of death, and we could not differentiate MI-related mortality with extraneous causes. This may underestimate the effect of asthma and long-term mortality because of smoking-related deaths in the exposed group.

## Conclusion

Although people with asthma were more likely to have a delay in their diagnosis following an STEMI but not an nSTEMI compared to the general population, were more likely to have a delay in reperfusion therapy and were much less likely to receive beta blockers following an STEMI or nSTEMI, there was no difference in the prescriptions of other secondary prevention medications. None of the differences in presentation or management were associated with an increase in mortality in people with asthma compared to the general population, suggesting that the relative underuse of beta blockers in people with asthma is appropriate.

## Supplementary Material

Supplementary material
