# Supplementary material for: Presentation, management and mortality after a first MI in people with and without asthma: A study using UK MINAP data
Source: Chron Respir Dis. 2017 Apr 10;15(1):60–70. doi: 10.1177/1479972317702140 (PMC5802653; doi:10.1177/1479972317702140)
Supplement: Supplementary material [file MI_Asthma.pdf]

Table S1: Sensitivity Analyses STEMIs (only patients who were non-smokers)

*Adjusted for age, sex, smoking, year of admission, diabetes, CVSD, CRF, CCF, PVD, low LVEF, previous angina, previous PCI,

STEMIS	No asthma	Asthma	Total
	32,699 (91.4%)	3,076 (8.6%)	35,775
	**% in No asthma**	**% in Asthma**	**Fully Adjusted OR (95% CI)**
**Diagnosis and admission**			
**Diagnosis Delay***	n=2,407 (7.4%)	n=3,076 (10.5%)	1.39 (1.07 – 1.81)
**Admission to CCU***	82.7%	81.3%	0.99 (0.87-1.10)
**Invasive Procedures**			
**Delayed reperfusion***	N= 10,304 (40.2%)	N=1,047 (45.2%)	1.18 (1.09-1.29)
**Use of reperfusion***	n=24,776 (75.8%)	n=2,231 (72.5%)	0.94 (0.80 – 1.11)
**Secondary Prevention**			
**Discharge on B-blocker***	n=24,849 (76.0%)	n=1,399 (45.5%)	0.24 (0.21 – 0.28)
**Discharge on aspirin* n=34,173**	n=26,491 (84.8%)	n=2,418 (82.6%)	0.97 (0.78 – 1.21)
**Discharge on clopidogrel* n=34,173**	n=19,935 (63.8%)	n=1,785 (61.0%)	1.05 (0.87 – 1.27)
**Discharge on ACEi* n=34,173**	n=24,639 (78.9%)	n=2,253 (77.0%)	1.11 (0.91 – 1.35)
**Discharge on Statins* n=34,173**	n=26,359 (84.4%)	n=2,431 (83.1%)	1.17 (0.92 – 1.49)
**Mortality**			
**In-Hospital Mortality****	n=1,453 (4.4%)	n=149 (4.8%)	1.03 (0.62 – 1.71)
**180-Post Discharge Mortality*** n=34,170**	n=2,314 (7.4%)	n=282 (9.6%)	1.05 (0.76 – 1.46)

previous CABG, family history of MI, treatment for hypertension, treatment for hyperlipidaemia, treatment with antiplatelets, tachycardia and hypotension at admission

****** Adjusted for ***,** diagnosis delay and use of reperfusion.

*******Adjusted for ******, and β-blocker, aspirin, clopidogrel, statin and ACEi at discharge.

Table S2. Sensitivity Analyses nSTEMIs

nSTEMIs	No asthma	Asthma	Total
	46,342 (89.5%)	5,846 (10.5%)	52,188
	**% in No asthma**	**% in Asthma**	**Fully Adjusted OR**
**Diagnosis and admission**			
**Diagnosis Delay***	n=17,772 (38.4%)	n=2,595 (44.4%)	1.04 (0.92 – 1.18)
**Admission to CCU***	36.7%	33.0%	0.89 0.84-0.95
**Invasive Procedures**			
**Elective PCI/CABG***	N=10,108 (21.8%)	N=1,053 (18.0)%	0.86 0.80-0.93
**Elective Angiography***	n=21,876 (47.2%)	n=2,470 (42.3%)	0.81 (0.70 – 0.93)
**Secondary prevention**			
**Discharge on B-blocker* n=50,873**	n=30,223 (66.9%)	n=1,964 (34.5%)	0.27 (0.24 – 0.30)
**Discharge on aspirin* n=50,873**	n=34,564 (76.5%)	n=4,272 (75.0%)	0.94 (0.82 – 1.07)
**Discharge on clopidogrel* n=50,873**	n=24,982 (55.29)	n=3,084 (54.2%)	1.04 (0.91 – 1.17)
**Discharge on ACEi* n=50,873**	n=28,518 (63.1%)	n=3,521 (61.9%)	0.97 (0.85 – 1.10)
**Discharge on Statins* n=50,873**	n=33,509 (74.2%)	n=4,140 (72.7%)	0.96 (0.84 – 1.11)
**Mortality**			
**In-hospital Mortality****	n=1,162 (2.5%)	n=153, (2.6%)	0.87 (0.46 – 1.65)
**180 days post-discharge mortality*** n=50,872**	n=5,626 (12.5%)	n=872 (15.3%)	1.05 (0.85 – 1.30)

*Adjusted for age, sex, smoking, year of admission, diabetes, CVSD, CRF, CCF, PVD, low LVEF, previous angina, previous PCI, previous CABG, family history of MI, treatment for hypertension, treatment for hyperlipidaemia, treatment with antiplatelets, tachycardia and hypotension at admission

****** Adjusted for ***,** diagnosis delay and use of angiograph.

*******Adjusted for ******, and β-blocker, aspirin, clopidogrel, statin and ACEi at discharge.
